# Physical and microbial root-zone factors underlying tomato wilt in long-term biofloc aquaponic systems

**DOI:** 10.1371/journal.pone.0349411

**Published:** 2026-05-18

**Authors:** Shima Rezaei, Margaret Otto, Qichen Wang, Wellington Arthur, Deepak Shantharaj, Kelly Sullivan, Daniel Wells, Neha Potnis, Brendan T. Higgins

**Affiliations:** 1 Biosystems Engineering, Auburn University, Auburn, Alabama, United States of America; 2 Entomology and Plant Pathology, Auburn University, Auburn, Alabama, United States of America; 3 Horticulture, Auburn University, Auburn, Alabama, United States of America; University of Agriculture Faisalabad, PAKISTAN

## Abstract

Biofloc aquaponics presents a sustainable approach to soilless crop production, but its long-term reliability is often compromised by root-zone disorders such as root rot and wilt. This study evaluated four media-based biofloc aquaponic designs over two years (encompassing two full fish production cycles and four tomato growing seasons) to investigate system failures, including plant wilting, necrosis, and the underlying physical and microbial stressors. In the first year, plant wilt was primarily linked to poor drainage and sludge accumulation in coupled systems, resulting in root-zone hypoxia and a 21% reduction in yield compared to decoupled systems. In response, system modifications were implemented in the second year, including the implementation of radial flow settlers upstream of filters and timer-controlled pumps, which improved oxygenation and solids management. These interventions reversed previous trends: coupled systems had > 45% higher yields than decoupled systems. However, wilting still occurred in a subset of plants during the second year. Root-zone analysis showed that wilted plants exhibited 65% lower dissolved oxygen levels than non-wilted, and a tendency for higher relative abundance of potential pathogens and parasitic organisms (*Pythium graminicola* and *Xiphinema rives*). In contrast, healthy root zones were associated with greater abundance of plant growth-promoting bacteria (PGPB), particularly taxa with known biocontrol potential. These findings highlight the importance of maintaining oxygen-rich root environments and effective solids management to support long-term aquaponic productivity.

## 1. Introduction

For thousands of years, soil has supported agriculture by providing structural stability and hosting a rich microbial ecosystem essential for plant growth. However, rising global populations and increasing pressure on freshwater resources have driven the need for more sustainable and space-efficient food production systems. Soilless cultivation technologies, particularly hydroponics and aquaponics, have emerged as promising alternatives that reduce water use, recycle nutrients, and enable high-yield production in urban and land-constrained settings [1–3]. Despite their environmental benefits and growing adoption, aquaponic systems continue to face challenges that limit their long-term reliability and scalability [4,5].

One of the most persistent issues is root stress, especially asphyxiation caused by prolonged waterlogging, insufficient oxygenation, and sludge accumulation in the root zone [6]. Unlike soil, which provides aerated pore spaces for gas exchange, aquaponic grow beds can become oxygen-depleted over time, especially in media-based systems with high organic matter buildup. This can severely impair root respiration, limit nutrient uptake, and weaken plant immune responses [7]. As reviewed by Garcia et al., hypoxia suppresses the expression of key defense-related genes, interferes with oxidative burst and structural barrier formation, and also disrupts the rhizosphere microbiome [8]. Intermittent irrigation strategies such as ebb-and-flow cycles have been employed to enhance oxygen availability in the root zone, but their effectiveness remains highly dependent on substrate maintenance and the efficiency of solid waste removal [9]. Maintenance of media-based grow systems is challenging in long-term crops such as the production of indeterminate tomatoes. Beyond direct physiological stress, high moisture and hypoxic conditions can shift root-zone microbial communities toward opportunistic pathogens, including *Fusarium*, *Pythium*, *Phytophthora*, and other oomycetes and fungi, while also enabling parasitic nematodes to exploit stressed roots, further impairing water and nutrient uptake [10,11]. Infections by *Pythium* spp. are commonly associated with root browning, soft rot, and cortical degradation, leading to wilting and plant death, particularly under conditions of high moisture, organic loading, and elevated temperatures [12–14]. Several aggressive species, such as *P. graminicola*, *P. myriotylum*, and *P. aphanidermatum*, have been frequently reported in hydroponic systems lacking optimal environmental control [15–17]. Despite these challenges, the mechanisms underlying root-zone dysfunction in aquaponics remain poorly understood. Previous studies have reported inconsistent disease suppression outcomes, with aquaponic systems showing variable performance compared to hydroponics [18]. Surveys of commercial operations in the United States and Europe further highlight persistent challenges in disease diagnosis and management, compounded by the dual-host nature of aquaponic systems [19–22]. Therefore, a clearer understanding of root-zone physical and microbial dynamics is needed to improve system reliability and long-term productivity.

To address these gaps, this study examined four media-based biofloc aquaponic system designs over two years, encompassing two full fish production cycles and four cherry tomato growing seasons. During system operation, wilting symptoms were observed in the fall trial of the first year, particularly in systems with higher fish densities and visible solid waste accumulation. These observations motivated a targeted investigation into the physical and microbial conditions of the root zone. In the second year, the system design was modified to improve solid waste removal and enhance root-zone oxygenation, including the installation of radial flow settlers for sludge separation and the use of timer-controlled pumps to maintain consistent ebb-and-flow cycles. This study aimed to identify the root-zone conditions contributing to plant wilt and reduced yields in media-based aquaponics by monitoring dissolved oxygen levels and characterizing microbial communities in the rhizosphere of symptomatic and asymptomatic plants.

## 2. Materials and methods

### 2.1. Experimental design

S1 Fig presents the four media-based biofloc aquaponic designs tested in this study: (1) dark-coupled (DC), (2) light-coupled (LC), (3) dark-decoupled biofloc (DD), and (4) light-decoupled biofloc [23]. The specific nature of these treatments has been described previously [24]; in brief, the coupled systems (DC and LC) operated with continuous water recirculation from the fish tank to the hydroponic grow bed at a flow rate of 9 L/min, corresponding to a hydraulic retention time (HRT) of approximately 80 minutes in the fish tank. This continuous flow mimicked recirculating aquaculture systems [20], with the media-filled grow bed serving as both a solids trap and a biofilter for nitrification and organic matter breakdown. In contrast, the decoupled systems (DD and LD) operated in a semi-continuous mode. Water was pumped from the fish tanks to the hydroponic beds twice per day for 3 minutes per cycle (25 L per cycle), resulting in a fish tank HRT of around 14 days. To maintain a stable volume of 700 L, fresh water was added daily at a rate of ~50 L, consistent with biofloc system water exchange rates reported in previous studies [25]. The extended HRT in these systems enabled the fish tanks to function as primary biofloc reactors, where internal microbial activity facilitated nitrogen mineralization and organic matter degradation, while also contributing supplemental nutrition for tilapia [26]. In the ‘dark’ treatments, fish tanks were painted and covered with dark fabric to prevent light exposure.

Each treatment was replicated three times in a randomized complete block design and operated in a greenhouse at the E.W. Shell Fisheries Station, Auburn, Alabama. Details of the greenhouse setup and weather conditions have been described previously [24,27]. All experimental protocols associated with tilapia production were approved by the Auburn University Institutional Animal Care and Use Committee (IACUC #22-4099). To prevent contamination of the fish fillet tissue with anesthetics, fish were sacrificed by decapitation and pithing, ensuring a quick death to minimize suffering. Only a few fish were sacrificed for biochemical analysis (not reported in the present study) and the rest were sold to local markets at the end of the study. During the first year, solids removal was achieved using two-layer foam filters (Pentair PF7 and PF17C), and water was pumped continuously to the grow beds in the coupled systems. In contrast, the decoupled systems pumped water from the fish tank to the grow beds using timer-controlled pumps set to operate twice daily for three minutes per cycle (9 L·min⁻¹). In addition, the decoupled systems recovered grow bed drainage water in a sump which recirculated water to the grow bed 8 times per day (10 minutes per cycle at 9 L·min⁻¹). Each grow bed employed a media-based hydroponic configuration using pea gravel (0.5–1.5 cm diameter) and featured a bell siphon to facilitate intermittent flooding and draining, promoting root zone aeration [28]. In the second year, solids management was upgraded by placing a 110-L radial flow settler (RFS) upstream of the foam filter (Pentair PF7 only) and integrating 30-minute on/off pump cycles in the coupled systems to maintain consistent ebb-and-flow irrigation. The pump cycling was used because the bell siphon became unreliable for maintaining ebb and flow once solids accumulated in the pore space of the media bed. A full description of the system layout, equipment, and modifications can be found in our earlier work [24,29].

### 2.2 Aquaponics system operation and maintenance

All systems were operated for around 10 months each year, covering two cherry tomato (cv. *Favorita F1*) production cycles (spring and fall) and one full Nile tilapia grow-out cycle per year. Because wilt symptoms occurred only during the fall seasons, the present study focuses on the fall trial of each year. The fall tomato trials were conducted from September 17 to December 11 (Year 1) and from July 25 to November 14 (Year 2). After each tomato production cycle, grow beds were manually cleaned to remove accumulated sludge. Potassium, a nutrient commonly deficient in aquaponic and aquaculture systems [30], was supplemented daily through the make-up water to maintain a target concentration of 100 mg/L in the fish tanks [31]. In Year 1, each fish tank was stocked in March with 40 all-male Nile tilapia fingerlings (~20 g), sourced from existing raceways at the E.W. Shell Fisheries Station. Fish were grown to market size (~500 g) over eight months and harvested in November. In Year 2, tanks were stocked with 40 tilapia fingerlings (~130 g) and harvested at ~430 g. Fish were fed twice daily, once in the morning (7–8 am) and once in the afternoon (1–2 pm). During the fall tomato trials, fish received Cargill Triton-3603 feed (36% crude protein, 6% crude fat, 4% crude fiber, 1% phosphorus) at a rate of 1.5–2% of body weight, adjusted based on water temperature, observed consumption, and growth determined by monthly fish sampling [32,33]. Water quality was monitored regularly. pH readings were taken daily from the fish tanks using a Bluelab MetCombo meter, and pH was adjusted as needed to maintain values near 7.0 using either 3 M hydrochloric acid or calcium carbonate [34,35]. Dissolved oxygen (DO) and temperature were recorded every other day using a Milwaukee MW600 portable DO meter. System operation protocols have been described in further detail in our previous publications [24,29].

### 2.3 Sludge measurements

Sludge was discharged from the bottom of each RFS unit twice weekly. The discharge volume was recorded on site, and representative samples were collected after thorough mixing. Total suspended solids (TSS) concentrations in the collected RFS sludge and in the fish tank were estimated via optical density (OD) at 550 and 680 nm. Calibration curves relating OD to TSS concentration were generated seasonally by centrifuging solids (3,000 g, 10 min), drying at 105 °C, and weighing, following the protocol by Bertrand-Krajewski [36]. To quantify biosolids accumulation within the grow beds, three gravel sample columns were taken per bed at the end of each tomato trial. Samples were rinsed and oven-dried to determine total solids.

### 2.4 Plant yield measurements

Ripe tomatoes were harvested throughout each growing cycle, and total fresh fruit weight was recorded per plant to determine cumulative seasonal yield. Each grow bed, containing five tomato plants, was considered an experimental unit (n = 3 per treatment). At the conclusion of each trial, shoot biomass was also harvested and weighed. Both yield and shoot mass were normalized per plant by dividing totals by the number of plants in each bed.

### 2.5 Root zone oxygen and stomatal conductance

A subset of plants from the Year 2 trial was further investigated to understand the underlying causes of wilting in certain tomato plants. Six wilting plants were identified in one LC and two DD systems. For each wilting plant, a healthy companion plant was identified in each grow bed for comparison of root zone conditions. For the following analyses, individual plants, rather than entire systems, were considered as the experimental unit. Dissolved oxygen (DO) levels near the root zone were measured using a Milwaukee MW600 portable DO meter, which was carefully positioned directly in the grow bed media near plant roots. The sensor remained in place for several days to capture representative DO levels under both drained and flooded conditions in symptomatic and asymptomatic plants. After each measuring period, the probe tip was cleaned (and membrane replaced if damaged). Stomatal conductance (gsw) was measured separately on upper and lower leaves using a LICOR LI-600 porometer, which employs a steady-state, open flow-through design. The device was clamped onto each leaf until stable readings were logged, then unclamped and moved to the next sample.

### 2.6 Microbial analyses

DNA was extracted from biomass pellets collected from various system compartments, including grow beds, root zones of symptomatic and asymptomatic plants, and RFS sludge. Root zone sludge included attached microbes but not root tissues; therefore, endophytes were not included in this analysis, which may limit interpretation of plant–microbe interactions. Extractions were performed using the FastDNA Spin Kit (MP Biomedicals, USA), and DNA concentrations were verified using the QuantiFluor dsDNA Quantification Kit (Promega, USA). Samples were submitted to MR DNA Laboratory (Texas, USA) for high-throughput sequencing. The 16S rRNA gene (primers 515F–806R) and 18S rRNA gene (primers 1391F and EukBr) were amplified and sequenced using the Illumina MiSeq platform. Reads were processed using the facility’s in-house bioinformatics pipeline, which included demultiplexing, denoising, and zero-radius operational taxonomic unit (ZOTU) classification based on a curated reference database. Sequencing data have been deposited in the NCBI Sequence Read Archive (SRA) under BioProject accession numbers PRJNA1333965 and PRJNA1335545.

To identify functionally relevant taxa associated with root rot and wilt, species-level annotations from the 16S and 18S rRNA datasets were screened for putative bacterial and fungal pathogens, plant-parasitic nematodes, and potentially beneficial microorganisms. Species detected in the dataset were cross-referenced against several authoritative databases, including BacDive, Phytopath, HealthyHydroponics, Nemaplex, and the EPPO Global Database, to determine their relevance to plant disease [37–42]. Automated text-matching tools were used to compare species lists with these databases to efficiently extract overlapping entries. Candidate pathogens were then manually verified through a targeted literature review to confirm host range, pathogenicity, and ecological traits. Representative sequences from critical taxa were re-evaluated through NCBI BLASTn to validate species identity based on percent similarity and statistical support. A similar two-stage strategy was applied to identify putitive plant growth promoting bacteria (PGPBs). Species were first matched to curated PGPB repositories, including PLaBAse [43] and the Auburn University rhizobacteria database [44]. Genera commonly associated with plant growth promotion, such as Bacillus, Streptomyces, Pseudomonas, and Bradyrhizobium, were screened, and species detected in those genera were subjected to a manual literature review to confirm documented roles in biocontrol, nutrient solubilization, or growth stimulation. Only taxa with peer-reviewed evidence supporting pathogenic or beneficial activity were included in the final analyses.

### 2.7 Statistical analyses

Each treatment was replicated across three independent aquaponic systems (n = 3), resulting in a total of 12 system-level experimental units. For system-level analyses (e.g., yield and RFS solids), the aquaponic system was treated as the experimental unit. For within-system comparisons (e.g., wilted vs. healthy plants), individual plants were treated as the experimental unit. For stomatal conductance measurements, 6 wilting plants were identified across 4 different aquaponics systems. All non-wilting plants in these same beds were also assessed for comparison (14 plants). For root-zone DO and microbial community analyses, four wilting plants (n = 4) and four non-wilting companion plants (from the same grow bed) were assessed. Although these sample sizes are limited, particularly for root-zone DO and microbial analyses, they reflect the number of observable wilt events within the system; therefore, statistically significant differences should be interpreted with appropriate caution. Differences in root-zone dissolved oxygen (DO), stomatal conductance, and microbial abundance between wilted and healthy plants were evaluated using a two-tailed independent groups *t*-test. RFS solids amount data were analyzed using multiple regression in R (v4.3.1), treating fish tank light exposure and system configuration (coupled vs. decoupled) as fixed effects, and sampling time as a random effect to account for repeated measures over time. Time-course ANOVA followed by Tukey’s HSD post hoc tests were conducted using the “car” and “agricolae” R packages to control for multiple comparisons among treatments, including RFS solids concentration, fruit and shoot mass, and microbial abundance (p < 0.05). Data normality were checked visually using Q-Q plots and homogeneity of variance was checked using Levene’s test and data transformations (e.g., natural log) were performed if needed to increase homogeneity of variance. Descriptive statistics (means and standard deviations) were calculated using Microsoft Excel.

## 3. Results and discussion

### 3.1. Sludge in the root zone environment can cause root asphyxiation

Although sludge can serve as a reservoir of nutrients and beneficial microbes, excessive accumulation in hydroponic beds was a major contributor to root zone degradation, leading to oxygen depletion and plant wilting across treatments, as observed throughout this study and previously reported in our earlier work [24,29]. Over the two-year study, four aquaponics system types were operated, encompassing one fish production cycle (from 50 g to 500 g on average) and two tomato growing seasons (spring and fall) per year. In both years, wilt, chlorosis, and stunted growth were observed primarily during the fall tomato trials, when larger fish, higher feed input, and increased organic loading placed greater stress on system hydraulics and solids management. In the fall trial of the first year, continuous recirculation in the coupled systems filtered suspended solids through the gravel media, and poor drainage and sludge buildup in the grow beds likely created anaerobic microzones around the root zone, which may have contributed to root asphyxiation and the observed wilting and stunted growth. Consequently, the coupled systems produced cumulative tomato yields that were 21% lower than those of the decoupled systems (Table 1), despite having higher nutrient levels and more nutrients falling within sufficient ranges for tomatoes [24].

**Table 1:** Fruit production and wet shoot mass per plant in the fall trial of the first and second year across the four aquaponics designs.

Treatments	Fruit production in year 1 (g)	Fruit production in year 2 (g)	Wet shoot mass in year 1 (g)	Wet shoot mass in year 2 (g)
LD	1750 ± 389^a^	1253 ± 762 ^b^	1841 ± 59 ^a^	1597 ± 252 ^b^
LC	1389 ± 93 ^a^	2239 ± 411 ^ab^	1327 ± 271 ^a^	2189 ± 317 ^b^
DD	1674 ± 102 ^a^	1543 ± 385 ^b^	1941 ± 98 ^a^	1887 ± 142 ^b^
DC	1324 ± 269 ^a^	3471 ± 511 ^a^	1825 ± 555 ^a^	3249 ± 428 ^a^

* Values are the means ± standard deviation of 3 treatment replicates. Means with the same letters were not statistically significant based on Tukey HSD on final points.

To address these issues, RFS and timer-controlled pumps were introduced in the second year to improve solid removal and maintain consistent ebb-and-flow irrigation, enhancing air exchange in the grow beds. These interventions reversed the trends caused by excessive sludge buildup observed in the first year. In the second-year fall trial, wilting symptoms in the coupled systems were minimal (observed only in one replicate of the LC system) and these systems significantly outperformed the decoupled ones in both growth and yield, producing 1,457 g more fruit mass (*p* < 0.0001) and 977 g more shoot mass (*p* < 0.001), respectively (Table 1). This was expected given the higher nutrient flows afforded by coupled operation as previously documented [24].

**Fig 1:**
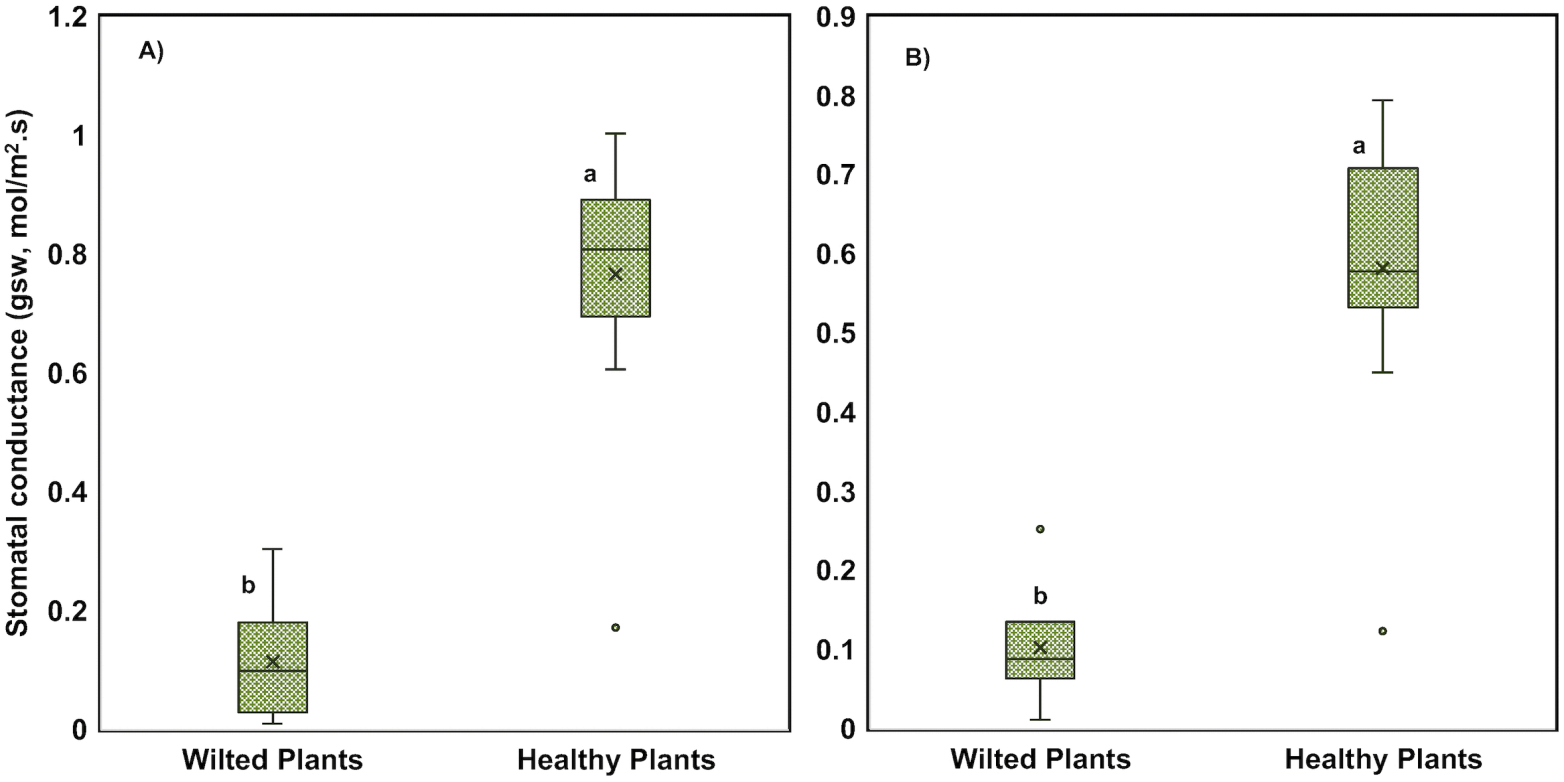
Stomatal conductance (gsw) for A) upper and B) lower leaves of wilted and healthy plants. Lowercase letters (a, b, c, etc.) indicate statistically significant differences between treatments (p < 0.05), n = 6 wilting plants and 14 non-wilting plants.

**Fig 2:**
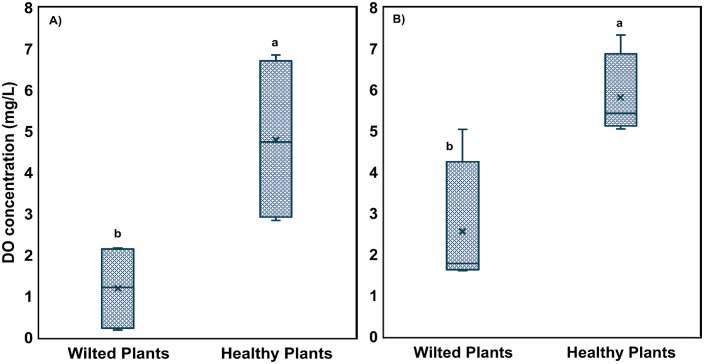
Root zone dissolved oxygen (DO) of wilted and healthy plants at A) drained and B) flooded irrigation intervals/levels. Lowercase letters (a, b, c, etc.) indicate statistically significant differences between treatments (p < 0.05), n = 4 plants for each group.

Despite these improvements in the coupled systems, root rot and wilting were observed in several decoupled systems, particularly the DD treatment. A total of 6 wilting plants were observed across DD and LC systems. Companion plants that were not wilting were selected from these same grow beds for comparison. Stomatal conductance measurements provided insight into the physiological stress of the wilted plants. Stomatal conductance was significantly lower in both upper and lower leaves of wilted plants (0.77 and 0.58 mol m⁻² s⁻¹, respectively) compared to healthy plants (1.11 and 1.10 mol m⁻² s⁻¹, respectively; *p* < 0.05) (Fig 1 and S1 Table), indicating impaired water uptake. This observation was consistent with DO measurements taken from the root zones. Root-zone DO was measured in 4 wilting and 4 non-wilting plants. The wilting plants under flooded conditions averaged 2.6 mg L⁻¹, which was significantly lower than the 5.8 mg L⁻¹ measured in non-wilting plants (*p* = 0.01, 2-tail independent group *t*-test) (Fig 2 and S2 Table). The grow bed’s drained period is the key time to enable root zone oxygenation, but even in the drained state, wilting plants had significantly lower DO (*p* = 0.02, 2-tail independent group *t*-test). Low oxygen availability around the roots likely reduced their ability to absorb water, which in turn may have contributed to partial stomatal closure [45]. While this response helps conserve water, it also limits photosynthetic activity and biomass accumulation, ultimately reducing plant productivity. Similar physiological responses to root hypoxia have been reported in other studies, where low DO levels led to reduced stomatal conductance and impaired growth [6,46].

**Fig 3:**
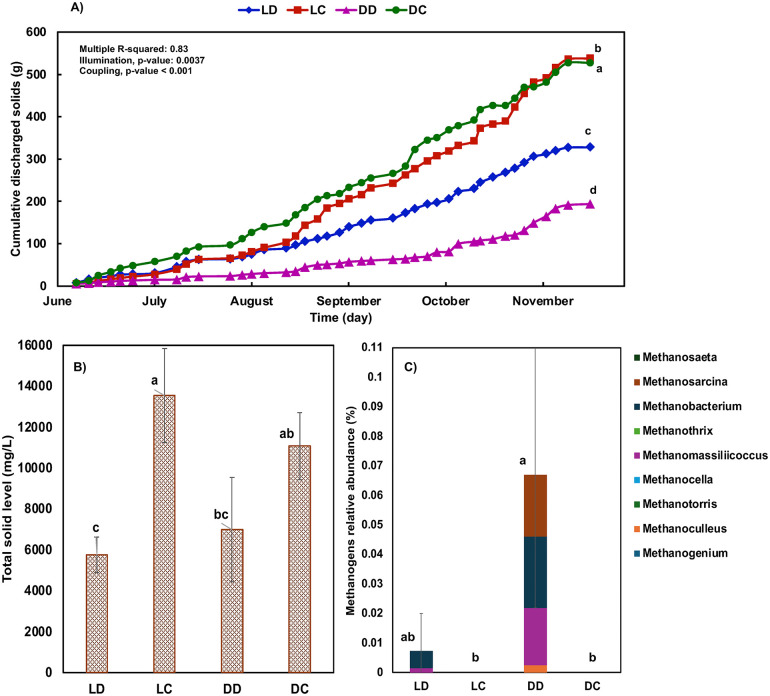
Sludge dynamics in aquaponics systems. **A)** Cumulative dried solids discharged from the radial flow settler (RFS) units over time across the four aquaponics designs: dark-coupled (DC), (2) light-coupled (LC), (3) dark-decoupled (DD), and (4) light-decoupled (23). **B)** Total solids accumulated in the grow beds in the fall trial. **C)** Relative abundance of methanogens in the RFS unit. Error bars represent standard deviation (n = 3). Lowercase letters (a, b, c, etc.) indicate statistically significant differences between treatments (p < 0.05).

It was initially believed that improving sludge removal through the incorporation of an RFS would universally improve the performance of aquaponic tomato production (and ideally eliminate problems with wilt). This was not the case. The performance of the RFS units varied among treatments (Fig 3A). Coupled systems, which circulated water through the clarifiers for 12 hours per day, achieved significantly greater solid removal compared to the biofloc-based decoupled systems, which operated their RFS units for only 6 minutes per day during irrigation cycles. Nevertheless, the coupled systems accumulated more sludge in the grow beds overall (Fig 3B), likely due to higher nutrient concentrations (no discharge system) and microbial growth [24,29]. Interestingly, although the decoupled systems maintained lower average sludge levels in their grow beds, some plants still experienced localized oxygen-deficient zones. This indicates that the spatial distribution of sludge, rather than total sludge accumulation alone, may play a critical role in driving root-zone hypoxia and plant stress. This was particularly evident near the clarifier outlets, where floating sludge occasionally escaped the RFS and settled on the grow bed surface. These sporadic sludge deposits created pockets of severe oxygen depletion, likely leading to localized root suffocation and wilting. Microbial community analysis further revealed differences between the clarifiers (Fig 3C). The decoupled systems, particularly the DD treatment, had a higher relative abundance of methanogens, strict anaerobes that thrive in low-oxygen environments and produce gases such as CO₂ and CH₄. Gas production may have contributed to sludge flotation and overflow, reducing settling efficiency in the DD clarifiers, which had the poorest solid removal performance among all treatments (Fig 3A). The floating sludge often settled near the hydroponic bed inlets, where it negatively affected the roots of nearby plants, causing localized oxygen depletion and wilting. As shown in S2 Fig, wilted plants had visibly brown, decayed roots, whereas healthy plants exhibited intact, well-developed root structures. These results underscore the critical role of consistent, well-timed, and spatially informed solid management strategies in aquaponic systems (not only to minimize total sludge accumulation but also to prevent uneven sludge distribution within grow beds) to prevent root rot and wilt and maintain a healthy rhizosphere. These findings suggest that total solids in the grow bed were not necessarily problematic (it was high in the most productive coupled system). Rather, it is the quality of the solid material that appears to be important. Long periods of stagnation in the clarifiers of the decoupled system, while theoretically good for solids settling, created undesirable anaerobic conditions. Thus inclusion of the RFS, while helpful in the coupled systems, may have had unintended negative consequences for decoupled system performance. In support of this conclusion, fruit yields and shoot mass were higher in the decoupled systems of year 1 (no RFS) than they were in year 2 (with RFS).

### 3.2. Sludge containing plant pathogens can damage roots and cause wilt

**Fig 4:**
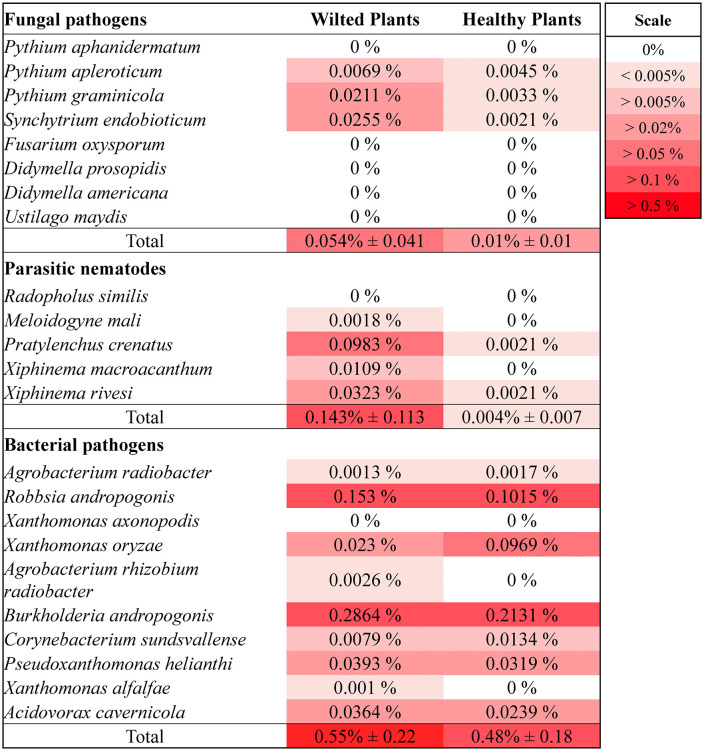
Relative abundance of putative fungal and oomycete pathogens, parasitic nematodes, and bacterial pathogens in the root zones of the wilted and healthy plants. Values are the means ± standard deviation of 3 treatment replicates.

As shown in Section 3.1, root-zone hypoxia associated with sludge accumulation was linked to reduced stomatal conductance and plant wilting, indicating that oxygen limitation was the primary driver of plant stress in this study. Waterlogged and oxygen-deficient conditions in aquaponic systems can also promote the proliferation of plant pathogens. Several microbial taxa known to include species that infect plant roots and vascular tissues were detected at higher relative abundances in the root zones of wilted tomato plants compared to healthy ones. These included fungal-like organisms (oomycetes and chytrids), plant-parasitic nematodes, and bacterial taxa with potential pathogenic traits (Fig 4). Taken together, these observations suggest that microbial dynamics acted as contributing or interacting factors within an already stressed root-zone environment, rather than as the primary cause of wilting.

#### 3.2.1 Fungal-like pathogens.

Wilted plants showed a higher cumulative relative abundance of putative fungal-like pathogens, most notably *Synchytrium endobioticum*, *Pythium graminicola*, and *Pythium apleroticum*, though the difference was not statistically significant (*p* = 0.12). *S. endobioticum*, the causal agent of potato wart disease, is a quarantine-listed chytrid fungus capable of producing persistent soil-borne sporangia that remain viable for 30–50 years [47]. While potato is its primary host, studies show it can infect other solanaceous crops, including tomato roots, potentially forming galls or subclinical infections [48]. Its elevated relative abundance in the wilted tomato roots (0.0255%) compared to the healthy ones (0.0021%) suggests a potential association with root stress under unfavorable conditions, although its direct role in tomato pathology remains uncertain. However, the difference was not statistically significant (*p* = 0.19).

*Pythium graminicola*, an oomycete widely associated with root rot and damping-off in cereals and solanaceous crops [49], was also more abundant in wilted roots (0.0211%) versus healthy roots (0.0033%), particularly in the DD system (not statistically significant, *p* = 0.11). This species produces motile zoospores that readily disperse in water, making it particularly well adapted to aquaponic environments. It infects fine roots and root hairs, causing water-soaked lesions, sloughing of the root cortex, and eventual necrosis. Infected tomato plants typically exhibit wilting, chlorosis, and reduced vigor, despite an intact shoot system [15,50], which is consistent with the symptoms observed in this study. Notably, *P. graminicola* was also detected at high levels (0.73% of relative abundance) in the fish tanks of DD systems, where it may have spread systemically and colonized the grow beds via circulating water. *Pythium apleroticum*, though less studied, belongs to the same clade and has been reported to cause fruit rot in tomato under laboratory conditions. It is considered weakly or opportunistically pathogenic but may contribute to disease in conjunction with other stressors [51,52]. Its detection exclusively in wilted root zones suggests a potential secondary or opportunistic role in exacerbating root decay, especially in systems with already compromised root health.

Interestingly, several potential PGPBs known for suppressing *Pythium* species were found at higher relative abundance in the root zones of healthy plants. While the differences were not statistically significant, the consistent presence of beneficial microbes in healthier root zones suggests an association with improved root conditions. However, this pattern may also reflect a response to favorable environmental conditions rather than a direct causal effect, as healthier roots may support or select for beneficial microbial communities. Notably, *Bacillus mycoides*, a spore-forming rhizobacterium recognized for producing phytohormones (e.g., IAA), solubilizing phosphates, and secreting antimicrobial metabolites [53], had a higher relative abundance in healthy roots (0.3016%) compared to wilted roots (0.0024%) (not statistically significant, p = 0.42). This species has shown antagonistic effects against soilborne pathogens, including *Pythium*, with some strains capable of inducing systemic resistance and emitting volatile compounds that inhibit oomycete growth [54]. For instance, strain SU-23 of *B. mycoides* has demonstrated complete suppression of *Pythium*-induced damping-off in cucumber [55]. Similarly, *Streptomyces* spp., which were also more abundant in healthy roots (e.g., *S. glaucescens* at 0.0438% and *S. scopuliridis* at 0.0286%) (not statistically significant, *p* = 0.31 and *p* = 0.5, respectively), are known for their broad-spectrum biocontrol properties. These filamentous bacteria produce antibiotics, lytic enzymes, and antifungal compounds that disrupt pathogen cell walls and are widely documented to protect tomato plants from root rot and damping-off [23]. Commercially used strains, such as *Streptomyces lydicus* WYEC108 (marketed as Actinovate), have been especially effective against *Pythium* species [56,57]. However, these interpretations are based on relative abundance patterns and do not confirm active pathogen suppression within the system.

#### 3.2.2 Parasitic nematodes.

Putative parasitic nematodes showed a trend toward higher relative abundance in wilted root zones, particularly *Pratylenchus crenatus*, *Xiphinema rivesi*, and *Xiphinema macroacanthum* (*p* = 0.09). *P. crenatus*, a root-lesion nematode, had a relative abundance of 0.0983% in wilted roots compared to 0.0021% in healthy roots (not statistically significant, *p* = 0.24). It penetrates cortical tissues and feeds endoparasitically, creating dark necrotic lesions and cavities that impair water and nutrient uptake. In tomato, infestation by *P. crenatus* is associated with pruned-back roots, reduced fine root density, and stunted, chlorotic shoots [58,59], which is consistent with the symptoms observed in the wilted LC and DD tomatoes. While lesion nematodes typically prefer soil environments, *P. crenatus* can persist in moist substrates as eggs or juveniles and may thrive in periodically drained aquaponic media. Its role in tomato yield losses has been documented, with studies showing up to 44% reduction under heavy infestations [60,61].

*X. rivesi*, a dagger nematode, is an ectoparasite that feeds on root tips using a long stylet and is known to vector nepoviruses such as Tomato ringspot virus (TomRSV) [62]. Although viral symptoms were not observed in this study, the feeding activity of *X. rivesi* alone can cause necrotic root lesions, tip swelling, and pruning of feeder roots, ultimately reducing water uptake and causing aboveground stunting [63]. Its relative abundance was 0.0323% in wilted roots and nearly absent in healthy ones (0.0021%) (not statistically significant, *p* = 0.41). *X. macroacanthum*, a genetically related species, shares similar feeding behavior and may contribute to root damage despite being less well studied. Parasitic nematodes were found in greater relative abundance in grow beds than in fish tanks (*p* = 0.047), and the coupled grow beds had higher levels of *X. rivesi* (*p* = 0.038), which may have contributed to wilt symptoms observed in the LC systems. Interestingly, the DC systems also exhibited elevated levels of *Xiphinema*, yet no disease symptoms were observed, possibly due to the absence of additional stressors such as root-zone oxygen deprivation, as observed by higher stomatal conductance of these plants (Fig 1). Thus, while potentially interacting with the tomato root systems, the presence of *Xiphinema* alone does not fully explain the wilting symptoms observed in these aquaponic systems.

**Fig 5:**
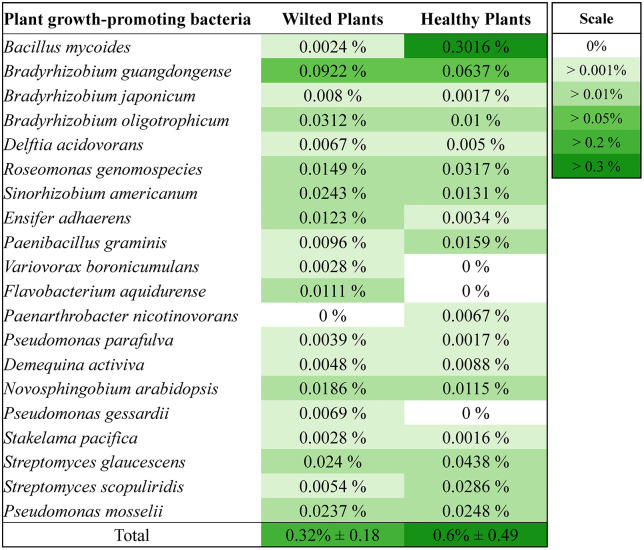
Relative abundance of putative plant-growth-promoting bacteria in the root zones of the wilted and healthy plants. Values are the means ± standard deviation of 3 treatment replicates.

Several of the putative PGPB genera detected, particularly *Bacillus*, *Pseudomonas*, and *Streptomyces*, are known to suppress plant-parasitic nematodes through both direct and indirect mechanisms (S3 Fig). These genera are among the most frequently reported nematode-antagonistic rhizobacteria in agricultural systems. *Bacillus* species, for instance, produce a variety of nematotoxic compounds, including crystal (Cry) proteins from *B. thuringiensis* that specifically disrupt nematode gut integrity. In addition, *Bacillus* spp. secrete extracellular enzymes such as chitinases and proteases that degrade nematode egg shells and cuticles, thereby inhibiting development and reproduction [64]. The *Bacillus* genus was detected at 0.3% relative abundance in the root zones of healthy plants, while its abundance in the root zones of wilted plants was 0.0013% (not statistically different, *p* = 0.42) (Fig 5 and S3 Fig). Additionally, certain *Streptomyces* strains have been shown to suppress populations of parasitic nematodes in tomato [65], which were also more prevalent in healthy root zones (not statistically different, *p* = 0.41) (Fig 5 and S3 Fig). As with other microbial observations, these patterns should be interpreted as associative and do not establish direct biocontrol activity within the system.

#### 3.2.3 Bacterial pathogens.

**Fig 6:**
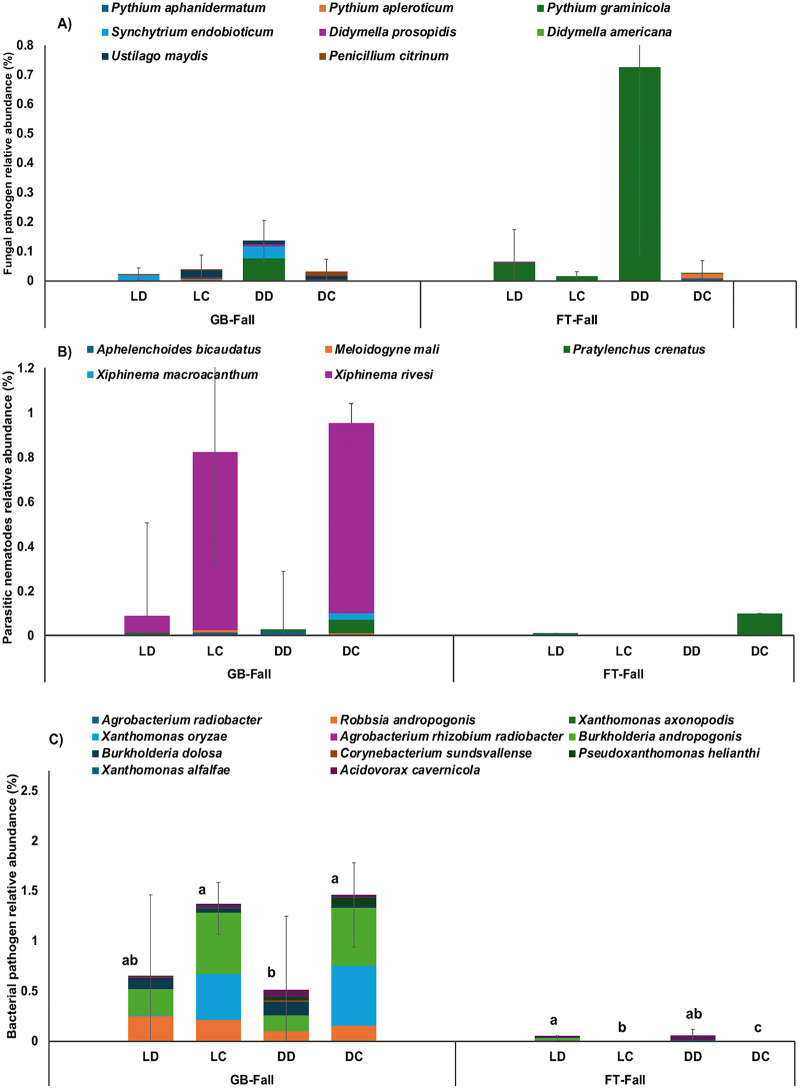
Relative abundance of (A) plant-pathogenic fungi and oomycetes, (B) parasitic nematodes, and (C) plant-pathogenic bacteria at the species level across fish tank [24] and grow bed [68] compartments. System designs: light-decoupled (23), light-coupled (LC), dark-decoupled (DD), and dark-coupled (DC). Error bars represent standard deviation (n = 3). Lowercase letters (a, b, c, etc.) indicate statistically significant differences between treatments (p < 0.05). Figures without letters were not statistically different from the others.

Several putative bacterial pathogens were also detected at higher relative abundances in the root zones of wilted plants, although the differences were not statistically significant. *Burkholderia andropogonis*, recently reclassified as *Robbsia andropogonis*, is a known foliar pathogen that causes leaf stripe, spot, and blight diseases in a range of hosts, including sorghum, areca palm, and ornamental plants. It carries a suite of virulence factors that enable aggressive colonization, particularly in wet environments where it can enter through stomata or wounds [66,67]. In this study, *B. andropogonis* and *R. andropogonis* were the most abundant pathogenic bacterial taxa, and were more prevalent in grow beds than in fish tanks (*p* < 0.0001) (Fig 6C), suggesting that the rhizosphere provided a favorable niche for their proliferation. with relative abundances of 0.439% and 0.315% in wilted and healthy roots, respectively. These taxa have not previously been confirmed as pathogens of tomato, but their broad host range and strong adaptation to moist conditions raise the possibility that they act as secondary or opportunistic colonizers, exacerbating root decay in already compromised plants or interfering with host defense responses. Despite less evidence of plant disease, nominally higher levels of putative bacterial pathogens were present in the highly productive coupled systems compared to the decoupled systems (*p* = 0.14). As with the parasitic nematodes, this fact suggests that bacterial pathogens were unlikely to be the primary drivers behind poor tomato growth performance in decoupled systems.

## 4. Conclusion

This study highlights the critical role of biosolids management in sustaining plant health and productivity in media-based aquaponic systems. Across two years of continuous operation, wilting and yield losses were primarily associated with oxygen-deficient root zones linked to poor drainage, sludge accumulation, and uneven solids distribution. Efforts to improve solids removal from fish tank water using an RFS were effective, but their downstream impact on tomato performance varied by system configuration. Solids removal clearly benefited the coupled systems, which produced substantially higher yields in the second year and exhibited minimal wilting. However, this intervention was associated with reduced yields and increased wilting in decoupled systems. The RFS units in decoupled systems exhibited characteristics consistent with anaerobic conditions, including elevated methanogen abundance, which may have contributed to localized root-zone stress, particularly near the water inlet. Low root-zone DO (as low as 1.4 mg L⁻¹) and reduced stomatal conductance (0.77 and 0.58 mol m⁻² s⁻¹) in wilting plants indicate impaired water uptake and physiological stress. These conditions likely created an environment favorable for opportunistic microbial colonization. Although not statistically significant, microbial analyses showed trends toward higher relative abundance of fungal-like pathogens and parasitic nematodes in wilted plants, while healthy root zones were associated with greater relative abundance of plant growth-promoting bacteria with known biocontrol potential. However, these observations are based on relative abundance data and should be interpreted as associations rather than evidence of direct functional activity. Overall, these findings indicate that physical root-zone conditions, particularly oxygen availability and sludge distribution, are primary drivers of plant performance, while microbial dynamics likely reflect secondary or interacting responses. As aquaponics systems scale toward commercial adoption, management strategies that maintain oxygen-rich root environments and minimize localized sludge accumulation will be critical. Importantly, increasing solids removal alone is not sufficient; system-specific design and operation must be considered. In this study, RFS implementation improved performance in coupled systems but was less effective in decoupled biofloc systems under the conditions tested.

## Supporting information

S1 Fig Schematic representation of the four aquaponic system types operated over ten months, encompassing fish growth from fingerlings to marketable size and two tomato production trials. The four system types include: (1) Dark-coupled (bacteria-dominant community), (2) Light-coupled (algae-dominant community), (3) Dark-decoupled (bacteria-dominant community), and (4) Light-decoupled (algae-dominant community).
(TIFF)

S2 Fig An example of (A) healthy and intact roots versus (B) rotten and brown roots. (TIFF)

S3 Fig Relative abundance of putative plant growth-promoting bacteria at the genus level across the root zones of healthy and wilted plants. (TIFF)

S1 Table Stomatal conductance in wilting and non-wilting plants. (XLSX)

S2 Table Root zone dissolved oxygen in wilting and non-wilting plants. (XLSX)
