# Supplementary figures and images for: Physical and microbial root-zone factors underlying tomato wilt in long-term biofloc aquaponic systems
Source: PLoS One. 2026 May 18;21(5):e0349411. doi: 10.1371/journal.pone.0349411 (PMC13183204; doi:10.1371/journal.pone.0349411)

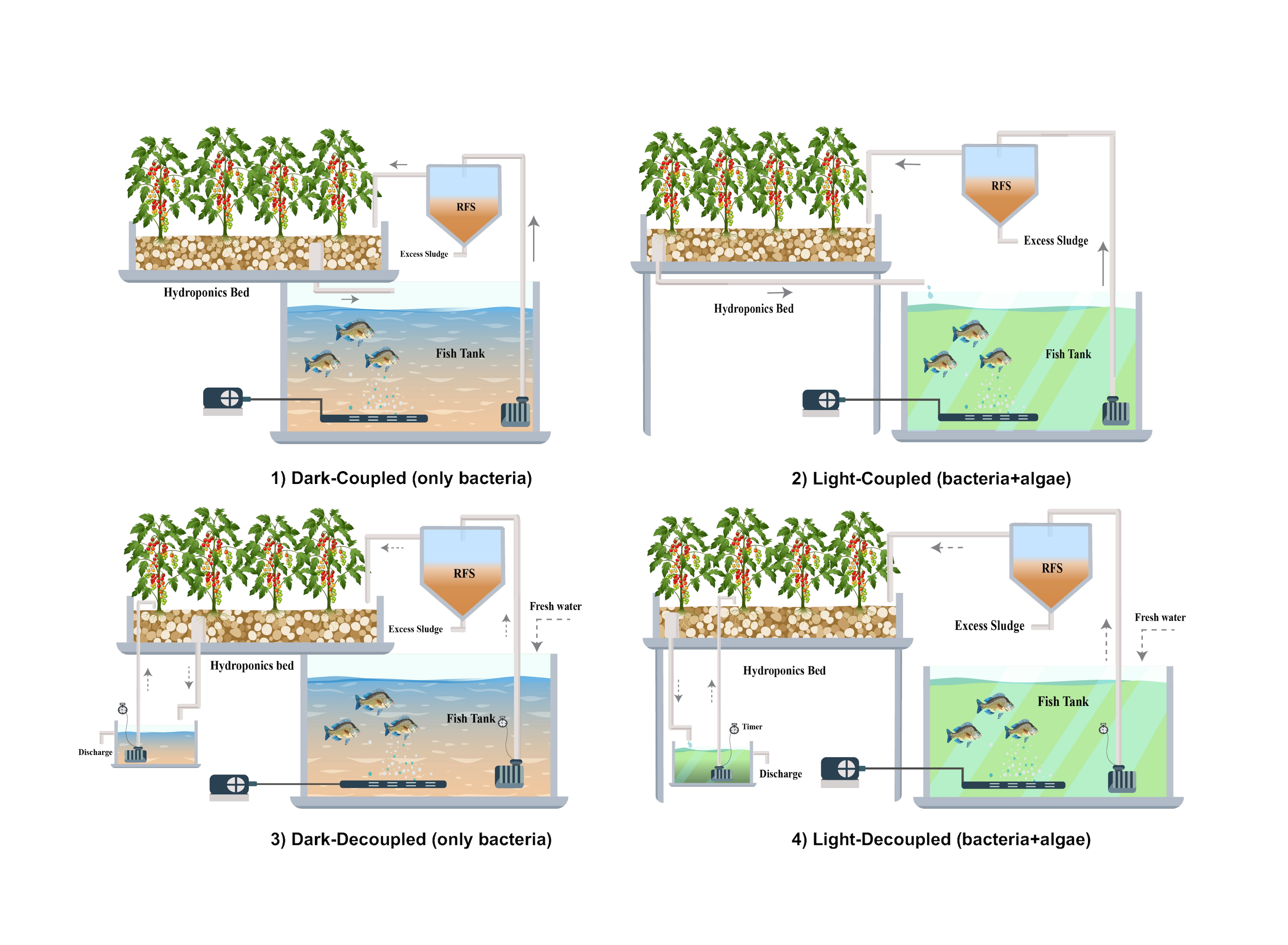

Supplement: S1 Fig — The four system types include: (1) Dark-coupled (bacteria-dominant community), (2) Light-coupled (algae-dominant community), (3) Dark-decoupled (bacteria-dominant community), and (4) Light-decoupled (algae-dominant community). (TIFF) [file pone.0349411.s001.tiff]

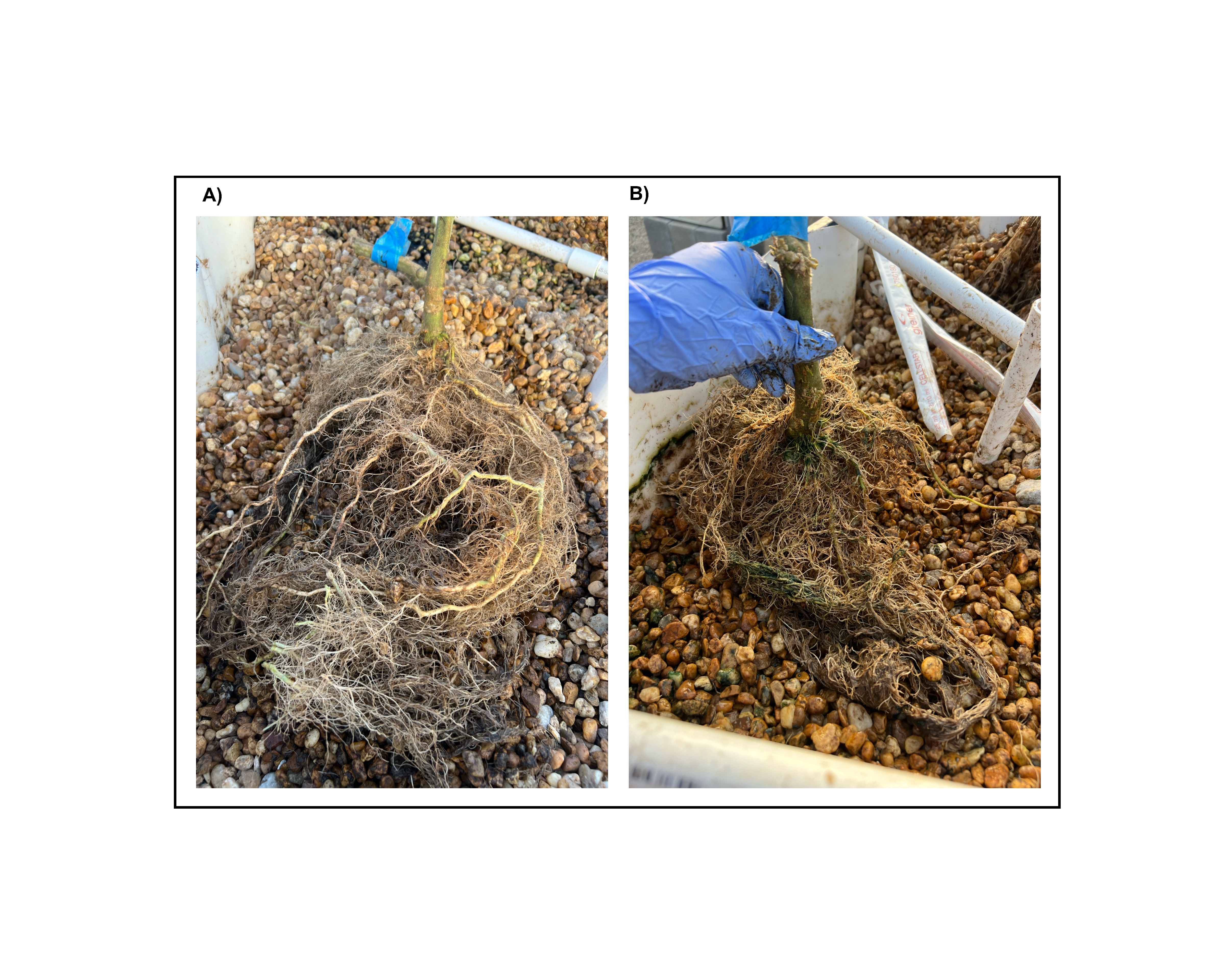

Supplement: S2 Fig — (TIFF) [file pone.0349411.s002.tiff]

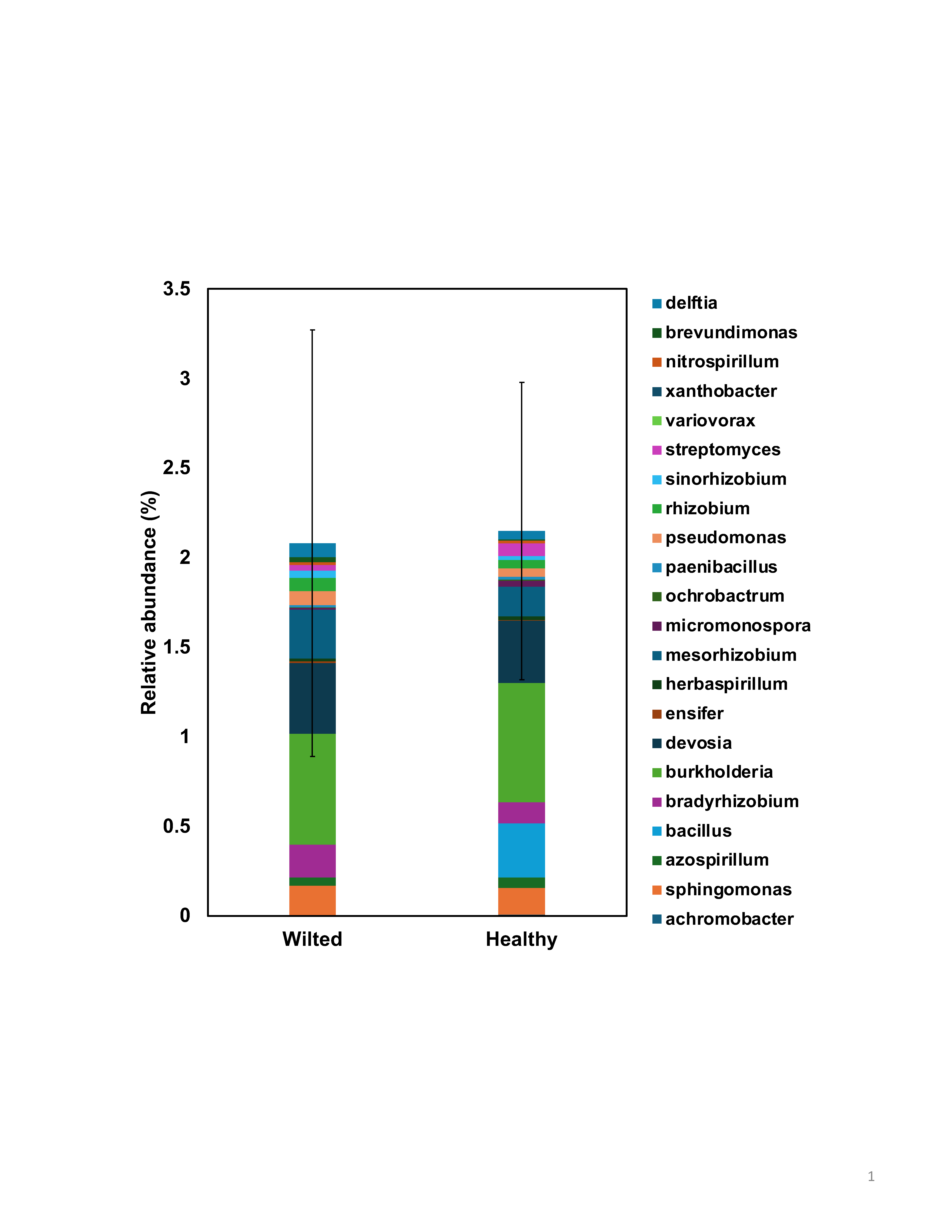

Supplement: S3 Fig — (TIFF) [file pone.0349411.s003.tiff]
